# Metformin versus sodium glucose co-transporters inhibitors as first-line for atherosclerotic cardiovascular disease: A meta-analysis

**DOI:** 10.12669/pjms.40.1.6982

**Published:** 2024

**Authors:** Amirah M. Alatawi

**Affiliations:** 1 Amirah M. Alatawi, Assistant Professor of Family Medicine, Department of Family and Community Medicine, Faculty of Medicine, University of Tabuk, Saudi Arabia

**Keywords:** Metformin, SGLT-2 inhibitors, First line, Atherosclerotic cardiovascular disease

## Abstract

There is growing evidence of prescribing sodium glucose co-transporters-2 inhibitor (SGLT-2) to patients with/at high risk of atherosclerotic cardiovascular disease as first-line (instead of metformin). This is the first meta-analysis to compare SGLT-2 inhibitors regarding the same. We aimed to compare SGLT-2 inhibitors and metformin regarding heart failure, acute coronary syndrome, and ischemic stroke. We systematically searched PubMed and Cochrane Library for relevant articles from the first article up to August 2022. The following keywords were used: Metformin, Salt glucose co-transporters inhibitors, SGLT-2 inhibitors, empagliflozin, dapagliflozin, canagliflozin, and first-line. The retrieved data were exported to an excel sheet detailing the author’s names, the country of origin of the study, the number of patients and control subjects, the study duration, and the total number of events in the interventional and exercise groups.

Out of 108 articles screened, only three studies fulfilled the inclusion criteria, a databased study, and two cohorts with 10309 events and 86487 patients. The present meta-analysis showed that SGLT-2 inhibitors had lower rates of heart failure (odd ratio, 1.51, 95% CI, 1.10-2.08) and myocardial infarction (odd ratio, 1.45, 95% CI, 1.08-1.96) than metformin with a similar rate of stroke (odd ratio, 1.03, 95% CI, 0.66-1.61). Significant heterogeneity was observed. Sodium-glucose co-transporter inhibitors-2 as first-line therapy showed a lower heart failure and myocardial infarction compared to metformin. No significant difference was found between the two drugs regarding ischemic stroke. Further larger studies comparing the adverse event are needed.

## INTRODUCTION

Metformin (a biguanide of herbal origin) is the most commonly prescribed drug for Type-2 diabetes mellitus. Since its first use in 1950, the drug was withdrawn due to lactic acidosis concerns after the withdrawal of two other biguanides in the United States of America. The drug was reintroduced in the year 1995 when proved safe.^1^ Metformin is the first-line oral hypoglycemic drug for the treatment of diabetes. However, it is also found to reduce certain cancers including colonic and breast cancer.^2^,^3^ Other uses of metformin are mortality reduction among obese patients admitted with COVID-19, polycystic ovary syndrome, and gestational diabetes^4^ In addition, metformin users showed a better cognitive function compared to non-users.^5^ Although metformin has been used as first-line therapy due to its benefits and higher safety profile, recent evidence suggested the use of novel drugs with cardio-renal protection including Sodium-glucose co-transporter 2 (SGLT-2) inhibitors and glucagon-like peptide agonists (GLP-1)^6^ SGLT-2 inhibitors were shown to reduce all-cause and cardiovascular mortality. myocardial infarction, body weight, and severe hyperglycemia with a lower risk of hypoglycemia.^7^ Early initiation of SGLT-2 inhibitors and GLP-1 agonists is recommended by unseating metformin and pushing it to the sidetrack.^8^ The American Diabetes Association recommended metformin as first-line and the European Association for the Study of Diabetes recommended SGLT-2 inhibitors and GLP-1 receptor agonists as first-line among patients with cardiovascular and renal disease.^9^,^10^ To the best of our knowledge, this is the first meta-analysis to compare metformin and SGLT-2 as first-line in the treatment of patients with Type-2 diabetes and higher/established cardiovascular risk. We aimed to assess metformin and SGLT-2i, as first-line therapy in Type-2 diabetes with established or higher cardiovascular risk.

## METHODS

### Articles selection according to PICOS

We searched PubMed, Cochrane Library, and Google Scholar from the first published article up to August 2022; we included, randomized controlled trials, prospective cohorts, retrospective studies, and case-control studies comparing metformin and SGLT-2i effects on heart failure, coronary artery disease, and stroke. Case series, case reports, and studies on animals were not included.

### Literature search and data extraction

We systematically searched the literature for relevant articles. Out of 108 articles retrieved, nine full texts were screened, and three studies were included in the meta-analysis (one database analysis and two prospective cohorts). The following keywords were used: Metformin, Salt glucose co-transporters inhibitors, SGLT-2 inhibitors, empagliflozin, dapagliflozin, canagliflozin, and first-line. The retrieved data were exported to an excel sheet detailing the author’s names, the country of origin of the study, the number of patients and control subjects, the study duration, and the total number of events in the interventional and exercise groups. The quality of the included studies was assessed using the New Castle Ottawa Scale.^11^

**Fig.1:**
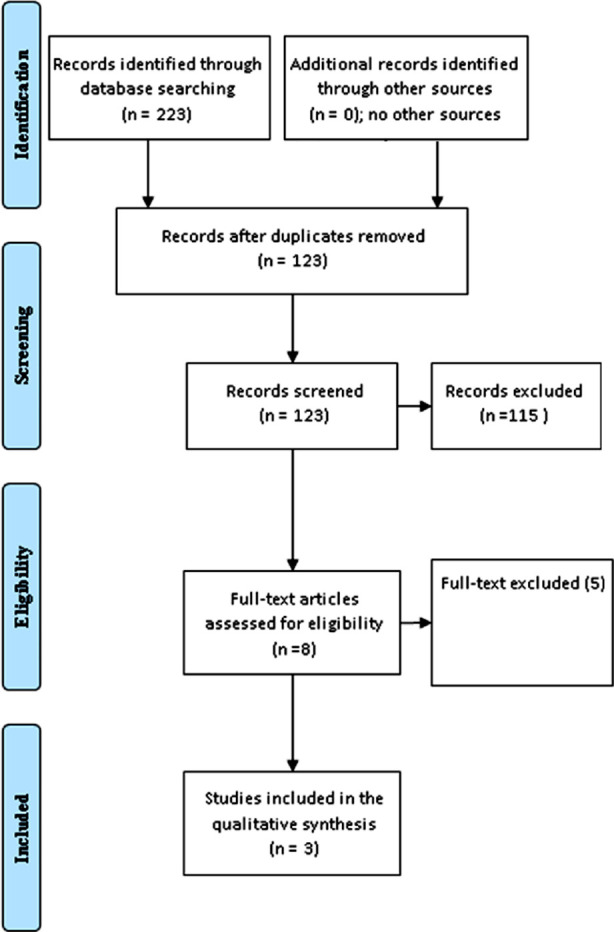
A comparison between metformin and SGLT-2 inhibitors regarding atherosclerotic cardiovascular disease.

**Table-I:** A comparison between metformin and salt-glucose cotransporters inhibitors-2 and first-line oral hypoglycemic medications.

Author	Country	Patients and duration	Metformin	SGLT-2 i	Results
Chen et al. 2020^12^	Taiwan	Database,12 months	8023/39920	278/1100	Higher among SGLT-2
Fralick et al. 2021^13^	USA	The observational study,147-213 days	996/9964	757/9964	Comparable efficacy
Shin et al. 2022^14^	USA	Prospective cohort, 7 years	172/17 226	83/8613	Comparable efficacy
Heart failure					
Chen et al. 2020^12^	Taiwan	12 months	5029/39920	69/1100	Higher among SGLT-2
Fralick et al. 2021^13^	USA	147-213 days	996/9964	807/9964	Comparable efficacy
Shin et al. 2022 14	USA	7 years	172/17 226	69/8613	Comparable efficacy
***Myocardial infarction***					
Chen et al. 2020^12^	Taiwan	12 months	2634/39920	38/1100	Higher among SGLT-2
Fralick et al. 2021^13^	USA	147-213 days	996/9964	879/9964	Comparable efficacy
Shin et al. 2022^14^	USA	7 years	172/17 226	69/8613	Comparable efficacy

### Data analysis

We use the RevMan (version 5, 4) for data analysis, the data were dichotomous and entered manually to compare the effect of metformin and SGLT-2 inhibitors on heart failure, coronary artery disease, and stroke. The random effect was applied due to the significant heterogeneity. A P-value of 0.05 is significant.

## RESULTS

**Fig.2:**
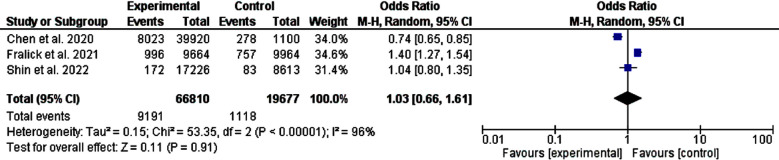
A comparison between metformin and salt-glucose cotransporters inhibitors-2 as first-line oral hypoglycemic medications (ischemic stroke).

In the present meta-analysis, we pooled three studies^12^-^14^ comparing metformin and salt-glucose co-transporters-2 inhibitors regarding heart failure, acute coronary syndrome, and ischemic stroke (a databased study, and two cohorts with 10309 events and 86487 patients). Two of the studies were published in the USA and one from Asia. No significant statistical difference regarding stroke (odd ratio, 1.03, 95% *CI*, 0.66-1.61, and P-value for overall effect, 0.91). Substantial heterogeneity was observed, *I^2^*, 96, P-value<0.001, and Chi-square, 53.35, Fig.2.

**Fig.3:**
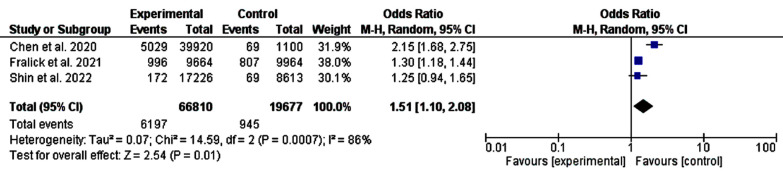
A comparison between metformin and salt-glucose cotransporters inhibitors-2 as first-line oral hypoglycemic medications (heart failure).

**Fig.4:**
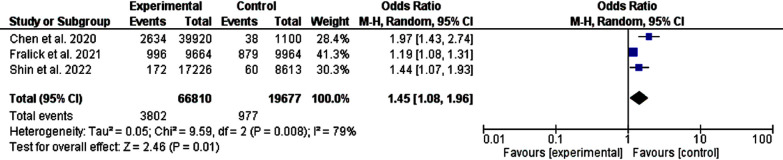
A comparison between metformin and salt-glucose cotransporters inhibitors-2 as first-line oral hypoglycemic medications (acute coronary syndrome).

Heart failure was lower among patients on SGLT-2 inhibitors compared to metformin (P-value, 0.01, chai-square, 14.59, and *I^2^* for heterogeneity, 86%, P-value for heterogeneity <0.001) and the acute coronary syndrome was lower among patients initiated SGLT-2 inhibitors as the first line (P-value, 0.01, chai-square, 9.59, and *I^2^* for heterogeneity, 79%, P-value for heterogeneity <0.001), Fig.3 and 4.

## DISCUSSION

The major goal of diabetes treatment is to reduce macrovascular complications, microvascular complications, and death.^15^ Although both metformin and sodium-glucose co-transporter two showed cardiovascular mortality reduction.^16^ However, no face-to-face meta-analysis was conducted.^17^ The present meta-analysis showed that SGLT-2 inhibitors had lower rates of heart failure (odd ratio, 1.51, 95% *CI*, 1.10-2.08) and myocardial infarction (odd ratio, 1.45, 95% *CI*, 1.08-1.96) than metformin with a similar rate of stroke (odd ratio, 1.03, 95% *CI*, 0.66-1.61). A recent study conducted in primary care found that 44% of patients with Type-2 diabetes had the coronary syndrome, heart failure, and kidney disease.^18^ Another interesting study showed that 27.7% of patients with Type-2 diabetes had undiagnosed heart failure.^19^ The current results imply that nearly half of patients with Type-2 diabetes qualify for treatments with SGLT-2 inhibitors. A recent Meta-analysis of randomized controlled trials showed that SGLT-2 inhibitors reduce heart failure hospitalization in people with diabetes by 32%.^20^ Importantly, SGLT-2 inhibitors were found to reduce incident atrial arrhythmias and sudden death.^21^ It is interesting to note that, no differences between empagliflozin, dapagliflozin, canagliflozin, and ertugliflozin in the reduction of heart failure hospitalization.^22^ Type-2 diabetes is a major independent risk for myocardial infarction and 20-30% of patients with myocardial infarction are suffering from Type-2 diabetes.^23^ The present results showed a lower incidence of myocardial infarction among SGLT-2 inhibitors compared to their counterparts taking metformin which can be a reasonable first-line therapy for patients with diabetes and myocardial infarction. The cardioprotective effects of SGLT-2 inhibitors may be due to lowering the blood pressure and weight, decreasing myocyte metabolism, and thus improving oxygenation.^24^ The association between SGLT-2 inhibitors and ischemic stroke is a matter of controversy. Some trials showed no association^25^, while others showed a non-significant increase.^26^,^27^ The current meta-analysis showed no significant difference between SGLT-2 inhibitors and metformin regarding ischemic stroke. The mechanism of increasing ischemic stroke might be due to hemoconcentration and hypovolemia.^28^ SGLT-2 inhibitors might be an appropriate choice for a patient with heart failure and myocardial infarction

### Sodium-glucose co-transporter inhibitors-2 and metformin fixed-dose combination

Combining different hypoglycemic medications with complementary mechanisms of action is the state of the art in Type-2 diabetes care. The combination of Ertugliflozin and metformin is an effective therapy for better glycemic control without increasing weight and lowering hypoglycemia risk.^29^ In addition, the fixed-dose combination improves adherence to medications. Fixed-Dose Combination of Canagliflozin and Metformin was effective in drug-naive patients and showed a reduced weight and blood pressure up to 26 weeks.^30^ A fixed-dose combination with empagliflozin was shown to be effective with minimal side effects.^31^ A fixed-dose combination of SGLT-2 inhibitors and metformin is cost-effective reducing medication burden and improving drug persistence.^32^

### Limitations

The small number of included studies and the significant heterogeneity observed limited this study.

## CONCLUSION

Sodium-glucose co-transporter inhibitors-2 as first-line therapy showed a lower heart failure and myocardial infarction compared to metformin. No significant difference was found between the two drugs regarding ischemic stroke. Further larger studies comparing the adverse event are needed.
